# Semi-quantitative indices of 2-[^18^F]FDG PET/CT in assessing cardiovascular and non-cardiovascular manifestations of IgG4-related disease and treatment response

**DOI:** 10.1186/s13550-023-00972-9

**Published:** 2023-03-17

**Authors:** Mingwei Chen, Charlene Yu Lin Tang, Warren Weng Seng Fong, Winnie Wing-Chuen Lam

**Affiliations:** 1 Lee Kong Chian School of Medicine, Nanyang Technological University, Singapore, Singapore; 2 Department of Nuclear Medicine and Molecular Imaging, Singapore General Hospital, Singapore, Singapore; 3 Department of Rheumatology and Immunology, Singapore General Hospital, Singapore, Singapore; 4 Duke-NUS Medical School, Singapore, Singapore

**Keywords:** PET, IgG4-related disease, Cardiovascular disease

## Abstract

**Background:**

IgG4-related disease (IgG4-RD) is a heterogeneous autoimmune disorder characterised by inflammatory lesions. Diagnostic imaging, such as 2-[^18^F]FDG PET/CT, is critical in evaluation of the disease, especially for potentially lethal cardiovascular manifestations. This retrospective study examines the usefulness of semi-quantitative parameters of 2-[^18^F]FDG PET/CT in monitoring IgG4-RD in patients with and without cardiovascular manifestations.

**Methods:**

Patients diagnosed with IgG4-RD who underwent a 2-[^18^F]FDG PET/CT scan were identified and classified based on presence or absence of cardiovascular disease. Clinical and laboratory data were extracted and compared to three 2-[^18^F]FDG PET/CT semi-quantitative parameters: maximum standardised uptake value (SUVmax), metabolic tumour volume (MTV), and total lesion glycolysis (TLG). Tissue-to-background blood (TBR) values were also evaluated for cardiovascular manifestations. These data were also compared in patients before and after receiving immunosuppressive therapy.

**Results:**

Forty-six patients identified were divided into an eight member subgroup of patients with cardiovascular manifestations, and a thirty-eight member subgroup of patients without. Patients with cardiovascular lesions were most frequently identified incidentally on imaging evaluation for other diseases (37.5%), with none presenting with chest pain or other cardiovascular symptoms. Ten patients with pre-treatment and post-treatment 2-[^18^F]FDG PET/CT scans demonstrated significant decreases in all semi-quantitative parameters, with no significant decrease in total IgG or ESR. The decrease in SUVmax, MTV, TLG following therapy was replicated in patients with and without cardiovascular manifestations.

**Conclusion:**

2-[^18^F]FDG PET/CT is an important investigation to determine all sites of involvement in this multisystemic disease and to rule out life-threatening cardiovascular manifestations even in the absence of symptoms. Semi-quantitative parameters such as SUVmax, MTV, TLG, and TBR are useful in assessing treatment response in patients. There are no serological substitutes that can quantify the extent of disease involvement like 2-[^18^F]FDG PET/CT.

**Supplementary Information:**

The online version contains supplementary material available at 10.1186/s13550-023-00972-9.

## Background

Immunoglobulin G4-related disease (IgG4-RD) is a rare heterogeneous autoimmune disorder characterised by tumefactive lesions with plasma cells positive for IgG4 [1]. It most commonly affects the pancreas, salivary glands, and periorbital tissues, but can also manifest in the cardiovascular system with lesions affecting the heart, pericardium, coronary arteries, thoracic aorta, abdominal aorta, and its branches. These cardiovascular manifestations are of particular concern because they may result in potentially lethal pseudotumours, inflammatory periaortitis, and pericarditis [1]. Diagnosis is complicated by its nonspecific and multisystemic presentations [2], with an important differential being lymphoproliferative disorders and other malignancies. Unlike such malignancies, IgG4-RD responds well to anti-inflammatory treatment [1].

Diagnostic testing modalities are, therefore, critical in evaluating IgG4-RD. 2-deoxy-2-[18F]fluoro-D-glucose positron emission tomography/computed tomography (2-[^18^F]FDG PET/CT) is a full-body scan that is useful in identifying sites of disease [3–6], monitoring response to anti-inflammatory therapy [5–11], and for biopsy site selection [2, 3, 7, 8, 12, 13]. However, semi-quantitative measures of 2-[^18^F]FDG PET/CT findings need to be further established. This is especially so in the key population of patients with cardiovascular lesions, given the diagnostic challenges for a potentially life-threatening manifestation. As serological markers of inflammation do not correlate with disease activity [7, 12, 14–16] and biopsy of cardiovascular lesions may be difficult, 2-[^18^F]FDG PET/CT findings are critical in assessing cardiovascular disease. This study aims to evaluate the utility of semi-quantitative parameters of 2-[^18^F]FDG PET/CT in monitoring IgG4-disease, including in patients with cardiovascular involvement.

## Methods

### Patients and study design

This is a non-interventional retrospective review from 1 January 2012 to 30 June 2021 of patients with IgG4-RD at the Department of Nuclear Medicine and Molecular Imaging of Singapore General Hospital. Patients were identified from Radiology Information System if they underwent a 2-[^18^F]FDG PET/CT scan with indications for IgG4-RD or had PET/CT scan findings suspicious for the disease. Corresponding patient information extracted from the clinical database include demographics, clinical presentation, histopathological reports, imaging reports, and laboratory data.

As per department protocol, and in accordance with the EANM guidelines, all patients fasted for a minimum of 4 h before the injection of tracer and had pre-procedural confirmation of blood glucose levels below 11 mmol/L. In patients who had known or suspected IgG4-RD, patients were placed on a combination of a high-fat, low carbohydrate diet starting from the day prior to the study and instructed for a prolonged fast of at least 12 h. Patients were scanned as per department protocol with delays of between 60 to 110 min post-injection of 2-[^18^F]FDG (246 MBq ± 40). The patients were scanned from vertex of the skull to the upper thighs on a lutetium oxyorthosilicate (LSO)-based PET/CT scanner with 64-slice CT (GE Discovery 690, Wisconsin, USA) and 70-cm transaxial field of view. PET images were reconstructed using an iterative ordered-subset expectation maximisation algorithm with 3 iterations, 24 subsets, and corrections for attenuation, scatter, and dead time. CT images were used for anatomical correlation, attenuation correction, and diagnosis (with intravenous contrast medium). PET data were acquired in 3-dimensional time-of-flight (TOF) mode, at 2 min per bed position and 5–6 bed positions per patient (25% overlap), depending on the patient’s size.

### Semi-quantitative analysis of PET/CT findings

Image analysis was carried out on a dedicated workstation using PACS (Carestream Vue PACS version 12). All 2-[^18^F]FDG PET/CT scans were read by two board-certified nuclear medicine physicians (WL and CT) independently, with discrepant interpretations resolved by consensus following simultaneous review and discussion. Abnormalities suggestive of IgG4-RD were recorded, these included visually abnormal metabolic uptake in locations unaccounted for by physiological 2-[^18^F]FDG distribution and corresponding to morphologically abnormal lesions on the CT done as part of the 2-[^18^F]FDG PET/CT study. Semi-quantitative analysis was performed for each lesion. The most commonly used PET semi-quantitative index, the maximum standardised uptake value (SUVmax) of each lesion was recorded using a standard volume of interest tool (VOI) tool, where the diameter of the VOI can be changed by the operator. The SUVmax of the VOI was calculated as (decay-corrected activity/tissue volume) divided by (injected dose/body weight). Volume-based indices such as metabolic tumour volume (MTV) and total lesion glycolysis (TLG) were also recorded. For MTV, a margin threshold as SUV of 2.5 was set to define the contouring margins around the target and boundaries were drawn to include all disease. Axial, coronal, and sagittal planes were all reviewed. Care was taken to exclude sites of physiological metabolic uptake, as well as benign or incidental lesions unrelated to IgG4-RD from the calculation of MTV. TLG was defined as the product of SUVmean and MTV. The SUV (maximal and mean), MTV, and TLG of the lesion were automatically demonstrated by the software. For patients with vascular involvement, the region of interest (ROI) was drawn in the axial plane and encompassed both the arterial wall and lumen. Volumetric mean venous blood pool activity (SUVblood) was measured with ROIs drawn simultaneously in 2-[^18^F]FDG uptake in the right atrium and inferior vena cava. As described in a previous study, SUVmax values of cardiovascular lesions were normalised by dividing the arterial SUVmax by SUVblood to generate tissue-to-background blood (TBR) values [18]. To compare semi-quantitative results between patients, the MTV and TLG for all 2-[^18^F]FDG-avid lesions were summated. For patients with cardiovascular presentations, the SUVmax of the most intensely 2-[^18^F]FDG-avid lesions (i.e. highest TBR and SUVmax values) was recorded.

### Statistical analysis

Descriptive statistics were reported in terms of frequencies and percentages for nominal data, mean ± standard deviation for parametric data, and median for non-parametric data. To compare demographic data in cardiovascular and non-cardiovascular subgroups, Fisher’s exact test was used. Spearman’s rho was evaluated to describe the relationship between laboratory and radiological results. To compare between subgroups of patients with and without cardiovascular disease, Shapiro–Wilk test was evaluated to assess for normality before applying the Mann–Whitney *U* test. A one-tailed paired *t* test was used to analyse the radiological findings in each patient before and after treatment. Statistical significance is established at *p* < 0.05. Statistical analysis was performed with SPSS Statistics (IBM SPSS Statistics for Windows, Version 28.0. Armonk, NY: IBM Corp).

## Results

### Demographics

Forty-six patients fulfilled the 2020 Revised Comprehensive Diagnostic Criteria for IgG4-RD as either definite, probable, or possible disease [17]. Twenty-eight males and eighteen females in the total patient population were divided into cardiovascular and non-cardiovascular subgroups, depending on the presence or absence of IgG4-RD lesions in the heart, blood vessels, or pericardium.

Thirty-eight patients of which twenty males and eighteen females formed the non-cardiovascular subgroup, while the cardiovascular subgroup composed of 8 male patients (Table 1). This difference in gender was statistically significant (*p* = 0.015), while the difference in distribution of ethnicities was not (*p* = 1.00). The mean age of diagnosis was higher in the cardiovascular subgroup at 60.9 years compared to 58.2 years, though this was identified to not be statistically significant (*p* = 0.262).

**Table 1:** Demographics of patients with IgG4-RD in cardiovascular and non-cardiovascular subgroups

Ethnicity	Number of patients	Percentage
Non-cardiovascular subgroup		
Chinese	30	78.90%
Malay	3	7.90%
Eurasian	2	5.30%
Caucasian	1	2.60%
Others	2	5.30%
Cardiovascular subgroup		
Chinese	8	100%

Gender	Number	Percentage
Non-cardiovascular subgroup		
Male	20	52.60%
Female	18	47.40%
Cardiovascular subgroup		
Male	8	100%

Twenty-eight male and eighteen female patients were diagnosed with IgG4-related disease. The cardiovascular subgroup comprised of eight male patients

### Initial clinical presentation

**Fig. 1:**
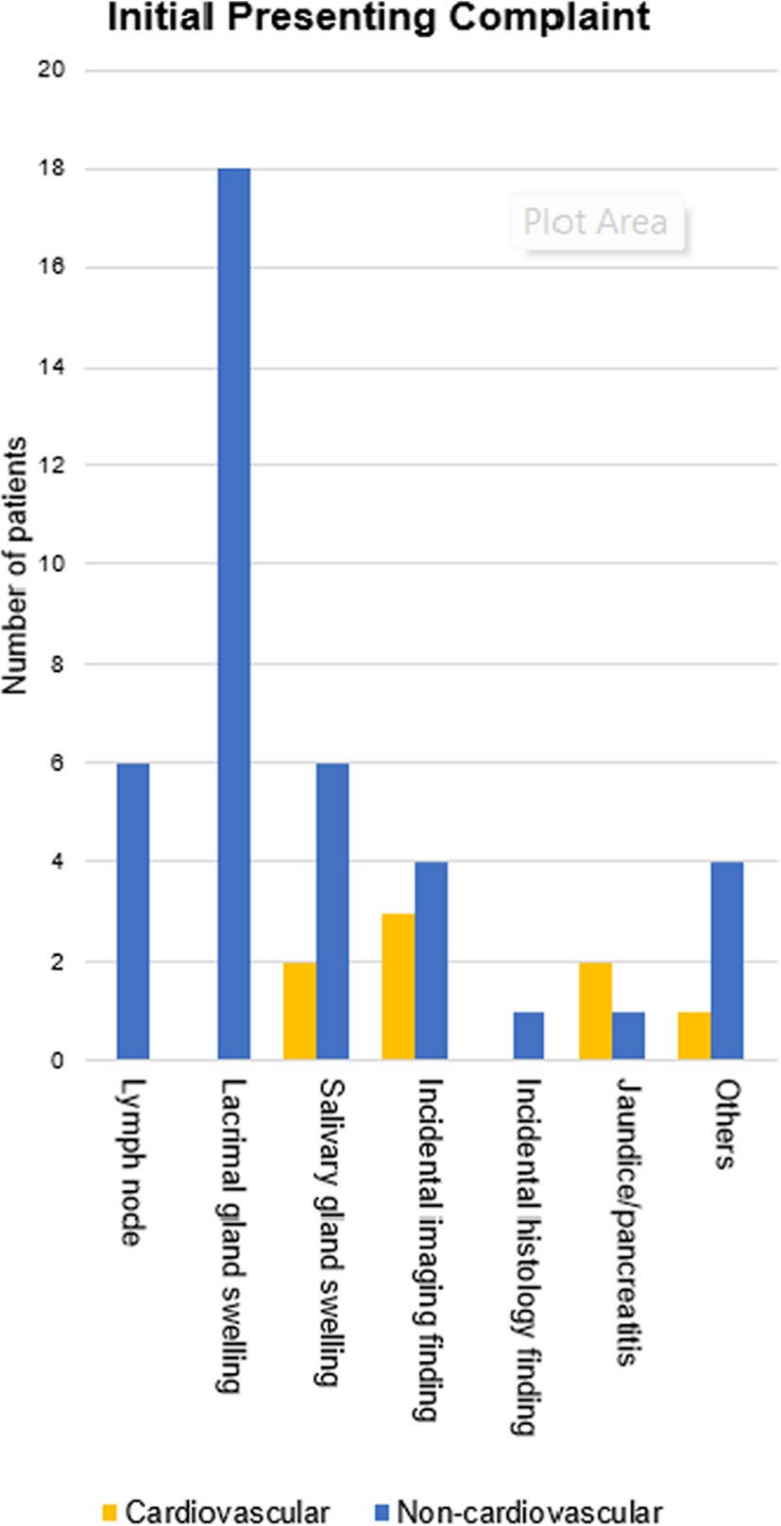
Initial presenting complaints of all patients diagnosed with IgG4-related disease. The most common presentation in all patients with IgG4-related disease was lacrimal gland or orbital gland swelling, followed by salivary gland swelling. Two patients presented with both lacrimal and salivary gland swelling.

Out of these 46 patients, the most common presentation was lacrimal gland or orbital swelling in 18 patients (39.1%), followed by salivary gland swelling in 8 patients (17.3%) (Fig. 1). Notably, 8 patients (17.3%) did not have symptoms related to IgG4-RD and were identified incidentally during radiological or histopathological evaluation for other diseases. Within the cardiovascular subgroup, IgG4-related disease was most often detected incidentally (37.5%) with no patients in this subgroup presenting with chest pain, dyspnoea, or other cardiovascular symptoms.

### Initial imaging and histopathological findings

Initial 2-[^18^F]FDG PET/CT scans identified 36 sites of organ involvement amongst the 8 patients in the cardiovascular subgroup, and 73 sites of organ involvement amongst 38 patients in the non-cardiovascular subgroup (Table 2).

**Table 2:** Sites of disease involvement in cardiovascular and non-cardiovascular subgroup

	Cardiovascular (number of sites of involvement)	Non-cardiovascular (number of sites of involvement)
Lymph node	6	24
Lacrimal gland	2	15
Submandibular gland	3	8
Parotid gland	2	7
Pancreas	3	4
Soft tissue (non-retroperitoneal)	1	4
Coronary artery	5	0
Retroperitoneal fibrosis	2	1
Aorta and other vessels	4	0
Breast	0	2
Prostate	1	1
Spleen	2	0
Uterus	0	1
Pleura	0	1
Kidney	1	0
Seminal vesicles	1	0
Liver	0	1
Sinuses	0	1
Colon	1	0
Pituitary gland	0	1
Pericardium	2	0
Thyroid	0	1
Larynx and glottis	0	1
No abnormalities on scan	1	3

109 sites of organ involvement were identified across 46 patients. Lymph nodes were the most commonly identified site of disease involvement

Cardiovascular involvement was identified based on lesions in the pericardium (*n* = 2), coronary arteries (*n* = 5), as well as aorta and other vessels (*n* = 4) (Table 3). In descending order of frequency, the sites of involvement in arteries are abdominal aorta (*n* = 3), iliac arteries (*n* = 3), followed by hepatic arteries, descending aorta, coeliac artery, renal artery, and right sided mesenteric vessels (*n* = 1 each). Patients with cardiovascular involvement had more extensive disease with a higher mean number of organs involved at 4.625 ± 3.20 compared to patients from the non-cardiovascular subgroup at 1.89 ± 1.20 (*p* = 0.049). In both groups, lymph nodes were the most frequently identified site.

**Table 3:** Cardiovascular manifestations of IgG4-related disease

Patient number	Cardiovascular manifestation
1	Pericardium
2	Descending aorta Abdominal aorta including coeliac, renal, common iliac arteries
3	Left hepatic artery Abdominal aorta including right and left common iliac arteries Right coronary artery
4	Right and left coronary arteries
5	Hepatic artery involvement identified on CTAP. FDG PET/CT scan performed after treatment demonstrated resolution of metabolic lesions
6	Right coronary artery
7	Right and left coronary arteries Pericardium Right internal iliac artery
8	Right and left coronary arteries Abdominal aorta including right sided mesenteric vessels

The cardiovascular manifestations included lesions in the pericardium, coronary arteries, as well as aorta and other vessels

Multiple organ involvement, defined as at least two organs with suspected IgG4-related disease, was more frequent in patients within the cardiovascular subgroup (75.0%) than in the non-cardiovascular population (52.6%).

### Comparing inflammatory biomarkers

For each patient, paired values of serum immunoglobulins (IgG4, or total serum IgG), inflammatory biomarkers (CRP, ESR), and semi-quantitative parameters on 2-[^18^F]FDG PET/CT (SUVmax, MTV, TLG) were compared. TBR values were also compared for cardiovascular lesions. Spearman’s rho identified no strong correlation between these variables (Table 4), though there was a moderate correlation between total IgG and ESR (*p* < 0.001) for all patients. For patients with cardiovascular disease, correlations between IgG4 and TBR, and between total IgG and TBR were not significant (*p* > 0.05).

**Table 4:** Correlation between serum immunoglobulins vs inflammatory markers and semi-quantitative parameters

	IgG4		Total IgG	
	Spearman’s rho	*p* value	Spearman’s rho	*p* value
ESR	− 0.043	0.862	0.740	0.000
CRP	0.363	0.203	0.279	0.334
SUVmax	0.179	0.402	0.374	0.072
MTV	− 0.181	0.396	0.416	0.043
TLG	− 0.193	0.367	0.463	0.023
TBR	− 0.685	0.202	0.686	0.201

Spearman’s rho evaluated the strength of correlation between total serum IgG and IgG4 with ESR, CRP, SUVmax, MTV, TLG, and TBR

### Comparing cardiovascular and non-cardiovascular subgroups

For cardiovascular and non-cardiovascular subgroups, Shapiro–Wilk test identified that all 7 variables were not normally distributed (*p* < 0.001 to 0.001). Therefore, to compare the extent of similarity between these two subgroups, the Mann–Whitney *U* test was applied. The two subgroups were significantly different for total serum IgG (*U* = 0.024), MTV (*U* = 0.006), and TLG (*U* = 0.006) but were not significantly different for IgG4 (*U* = 0.120), CRP (*U* = 0.940), ESR (*U* = 0.072), and SUVmax (*U* = 0.467).

### Comparing radiological findings pre- and post-treatment

Ten patients identified had repeat 2-[^18^F]FDG PET/CT scan following the initiation of treatment.. This group of 10 patients consisted of 5 patients with cardiovascular involvement, and 5 patients without cardiovascular involvement. Immunosuppressive therapies administered include, in order of decreasing popularity: prednisolone, mycophenolate mofetil, methotrexate, and rituximab.

All patients had a decrease in TLG after starting immunosuppressive therapy, with median treatment duration of 284 days. Representative images of the PET/CT scans are shown in the appendix (Additional file 1: Fig. S1, Additional file 2: Fig. S2, Additional file 3: Fig. S3), demonstrating a notable decrease in 2-[^18^F]FDG uptake following therapy. Overall, there was a marked decrease in TLG from a mean of 1066.61 ± 1265 in the pre-treatment group to 53.24 ± 39.1 in the post-treatment group (*p* = 0.0193). Similar statistically significant decreases were also observed across other semi-quantitative measures of SUVmax (*p* = 0.001), MTV (*p* = 0.019), and number of involved organs (*p* = 0.002), across both cardiovascular and non-cardiovascular subgroups (Table 5). Within the cardiovascular subgroup, there was a significant decrease in TBR before and after treatment (*p* = 0.002). While semi-quantitative measures reflected this change, laboratory indicators such as total IgG (*p* = 0.058) and ESR (*p* = 0.243) did not demonstrate a statistically significant change before and after treatment. There were insufficient post-treatment data for CRP and IgG4 to analyse their utility in reflecting response to treatment.

**Table 5:** Comparisons of semi-quantitative parameters and radiological findings before and after treatment

	Before therapy		During therapy		One-tailed *p* value
	Mean	Variance	Mean	Variance	
TLG					
Total	1066.61	1,778,964.88	53.24	1699.45	0.019
Cardiovascular subgroup	1974.15	1,926,531.95	67.00	1978.97	0.019
Non-cardiovascular subgroup	159.08	17,094.12	39.48	1371.45	0.037
SUVmax					
Total	8.74	23.80	2.96	3.56	0.001
Cardiovascular subgroup	9.46	46.61	2.60	1.31	0.035
Non-cardiovascular subgroup	8.02	5.65	3.32	6.37	0.004
MTV					
Total	321.56	147,654.62	30.40	571.32	0.019
Cardiovascular subgroup	589.66	150,410.42	36.12	578.80	0.017
Non-cardiovascular subgroup	159.08	17,094.12	39.48	1371.45	0.037
Number of lesions					
Total	4.60	7.16	1.40	0.49	0.002
Cardiovascular subgroup	6.00	10.00	1.40	0.30	0.012
Non-cardiovascular subgroup	3.20	1.20	1.40	0.80	0.027
TBR					
Cardiovascular subgroup	3.00	0.0956	1.524	0.066	0.002

	Before therapy		During therapy		
	Mean	Variance	Mean	Variance	One-tailed p value
Total IgG	30.51	509.58	10.65	3.17	0.058
ESR	49.20	2812.70	38.00	1568.50	0.243

Out of 10 patients with pre-treatment and post-treatment 2-[^18^F]FDG PET/CT scans, there were significant decreases in TLG, SUVmax, MTV, and number of lesions, but no significant decrease in total IgG and ESR. There was also statistically significant difference in TBR for the cardiovascular subgroup

## Discussion

This is the one of the largest retrospective studies to date exploring the utility of 2-[^18^F]FDG PET/CT in patients diagnosed with IgG4-related disease. The present findings provide evidence that semi-quantitative analysis with SUVmax, MTV, TLG, and TBR are useful parameters in the management of IgG4-RD. In this group of patients, the most commonly involved organs include lymph nodes, lacrimal glands, and salivary glands, which is unlike a previous local epidemiological survey which identified pancreas, lymph nodes, and bile ducts as the most common sites of disease [19]. This difference could be in part due to patterns of referrals between different departments, or a preference for magnetic resonance cholangiopancreatography or alternative imaging techniques in evaluating hepatobiliary disease. In our group of patients, the most commonly involved sites of disease tend to be organs that could be palpated or observed by the patient and physician. Diagnosing and monitoring disease involvement in anatomically deeper organs is more challenging. In particular, cardiovascular lesions are especially difficult to assess and yet are crucial to identify due to the possibility of life-threatening complications [20, 21]. In addition, these patients did not present with chest pain, dyspnoea, or other cardiovascular symptoms and instead largely presented with exocrine gland swellings or were identified incidentally. Therefore, given the nonspecific and multisystemic presentations, a non-invasive whole-body imaging such as 2-[^18^F]FDG PET/CT is crucial to characterise the extent of disease involvement and to rule out the possibility of life-threatening cardiovascular lesions when IgG4-RD is suspected. Our study has demonstrated that patients with cardiovascular involvement tend to have more extensive IgG4-RD.

There are no serological substitutes that can quantify the extent of disease involvement like 2-[^18^F]FDG PET/CT. While frequently employed as measures of disease activity, serological investigations such as CRP, ESR, and the immunoglobulin panel were largely not correlated with semi-quantitative findings on imaging. Only total IgG showed statistically significant correlation, but the strength of this correlation is moderate. Comparing the PET semi-quantitative indices, SUVmax is easily measured and is as such the most commonly used parameter in clinical practice. However, it measures the value from only a single voxel and is not representative of the whole disease metabolic burden. MTV is a measure of disease burden with high metabolic uptake. TLG combines both volumetric and metabolic information. Both these indices have been well studied and shown to be efficacious in a large variety of malignancies, and it has also been shown to be useful in non-malignant conditions that show metabolic uptake. Compared to SUVmax alone, MTV and TLG provide a better reflection of the global disease burden. This is evident in the comparison of the cardiovascular subgroup with the non-cardiovascular subgroup. The cardiovascular subgroup has more extensive disease with a higher number of organs involved, and this is similarly reflected in the volume-based indices TLG/MTV, but not appreciated by the SUVmax.

While semi-quantitative measures of 2-[^18^F]FDG PET/CT showed a statistically significant marked decrease following treatment, laboratory indices such as ESR, CRP, and the immunoglobulin panel did not demonstrate a similar statistically significant change before and after treatment. Our findings are consistent with prior studies demonstrating that conventional biomarkers of disease activity, ESR, CRP, and serum IgG4 levels, do not appear to correlate with the metabolic activity of IgG4-RD lesions, with conventional biomarkers showing conflicting results as a measure of disease and response to treatment.

With respect to treatment, previous studies have demonstrated that decreases in 2-[^18^F]FDG uptake and metabolic activity reflect response to therapy [2, 3, 5, 7, 10, 11, 14–16]. Our results further add to this growing body of evidence. Both SUVmax and volume-based indices such as MTV and TLG demonstrated significant decreases following initiation of immunosuppressive medications, and TBR demonstrated significant decreases in the cardiovascular subgroup as well. While there exist significant differences in total serum IgG, MTV, and TLG between the cardiovascular and non-cardiovascular subgroups prior to treatment, both subgroups demonstrated a significant decrease following treatment. This response to treatment was not reflected in total serum IgG or ESR. Furthermore, 2-[^18^F]FDG PET/CT scans have been shown to be particularly helpful for cardiovascular lesions as these lesions have decreased metabolic uptake after therapy but remain stable in size [7]. Limitations of this study include its retrospective study design which led to incomplete data pairs. The levels of proinflammatory biomarkers and immunoglobulin panels were not measured at all time points for all patients, leading to exclusion of these data sets from analysis. However, this was made up for by the relatively large study cohort, especially for a rare disorder. Future research with a prospective study design may lend further credence.

## Conclusion

This study highlighted the utility of 2-[^18^F]FDG PET/CT scans in assessing the extent of multi-organ involvement and disease burden in IgG4-related disease, especially in the key subgroup of patients with cardiovascular presentations. SUVmax is a simple useful parameter in monitoring response to immunosuppressive therapy. TLG and MTV in 2-[^18^F]FDG PET/CT allow for measurement of the disease burden and provide better assessment of response to treatment. These indices serve as good imaging biomarkers for IgG4-RD and can provide a potential avenue to guide the dosage and titration of immunosuppressants, which is currently done at the physicians’ discretion.

## Supplementary Information

**Additional file 1: Fig. S1:** Representative image for IgG4-RD patient with cardiovascular disease. There is a marked decrease in 2-[18F]FDG uptake in coronary artery lesions and thoracic lymph nodes before therapy (figures a, c, e, g) and after initiating prednisolone (figures b, d, f, h).

**Additional file 2: Fig. S2:** Contrast-enhanced reconstructed ECG-gated CT Angiogram images shows diffuse mural thickening around the entire right coronary (RCA) (figure a), left anterior descending (LAD) (figure b) and left circumflex (LCx) (figure c). A fusiform aneurysm is also seen in the proximal/mid RCA junction (figure a, red arrow). Large bilobed saccular aneurysm involving the distal part of the left main (LM) extending to the proximal LAD and proximal CX at the bifurcation (figure b and c, white arrow).

**Additional file 3: Fig. S3:** Representative image of IgG4-RD patient without cardiovascular disease. Note the decreased 2-[18F]FDG uptake in the left submandibular gland and right axillary lymph nodes before (figures a, c, e, g) and after therapy with prednisolone (figures b, d, f, h)

## Data Availability

The datasets used and/or analysed during the current study are available from the corresponding author on reasonable request.
