# Supplementary figures and images for: Semi-quantitative indices of 2-[18F]FDG PET/CT in assessing cardiovascular and non-cardiovascular manifestations of IgG4-related disease and treatment response
Source: EJNMMI Res. 2023 Mar 17;13:22. doi: 10.1186/s13550-023-00972-9 (PMC10023819; doi:10.1186/s13550-023-00972-9)

## Slide 1
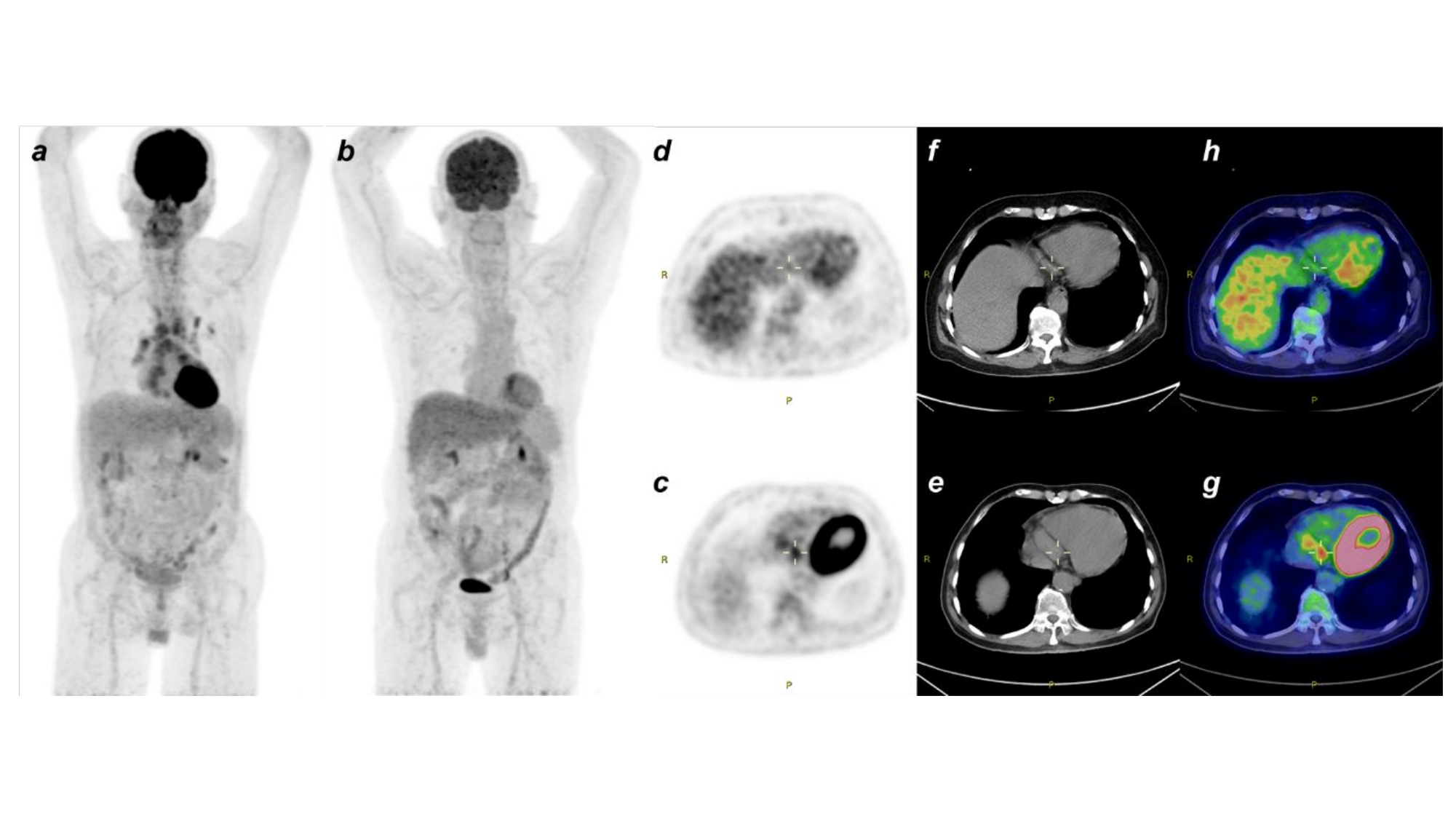

Supplement: Supplementary file 1 — Additional file 1: Fig. S1: Representative image for IgG4-RD patient with cardiovascular disease. There is a marked decrease in 2-[18F]FDG uptake in coronary artery lesions and thoracic lymph nodes before therapy (figures a, c, e, g) and after initiating prednisolone (figures b, d, f, h). [file 13550_2023_972_MOESM1_ESM.pptx]

## Slide 1
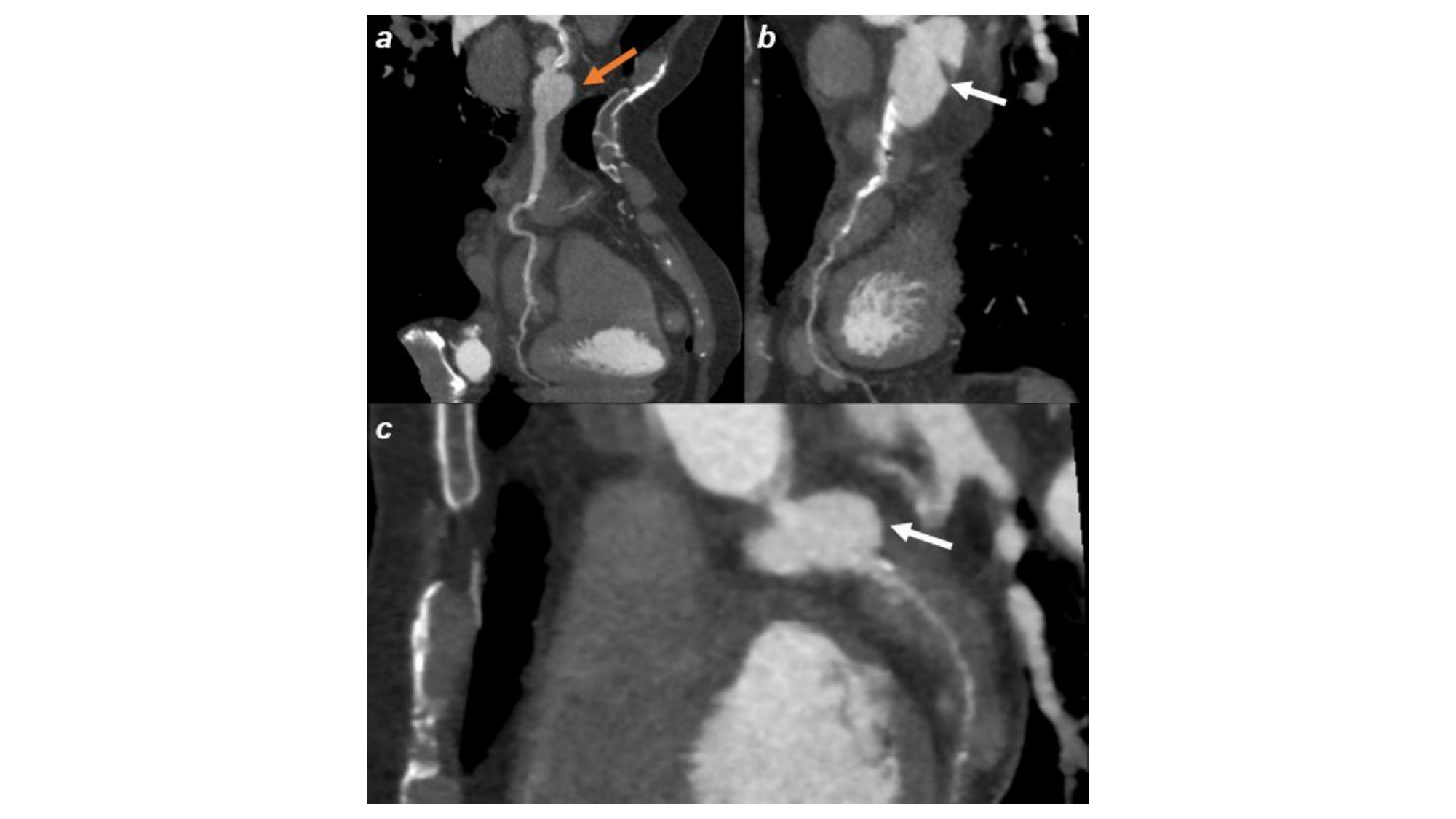

Supplement: Supplementary file 2 — Additional file 2: Fig. S2: Contrast-enhanced reconstructed ECG-gated CT Angiogram images shows diffuse mural thickening around the entire right coronary (RCA) (figure a), left anterior descending (LAD) (figure b) and left circumflex (LCx) (figure c). A fusiform aneurysm is also seen in the proximal/mid RCA junction (figure a, red arrow). Large bilobed saccular aneurysm involving the distal part of the left main (LM) extending to the proximal LAD and proximal CX at the bifurcation (figure b and c, white arrow). [file 13550_2023_972_MOESM2_ESM.pptx]

## Slide 1
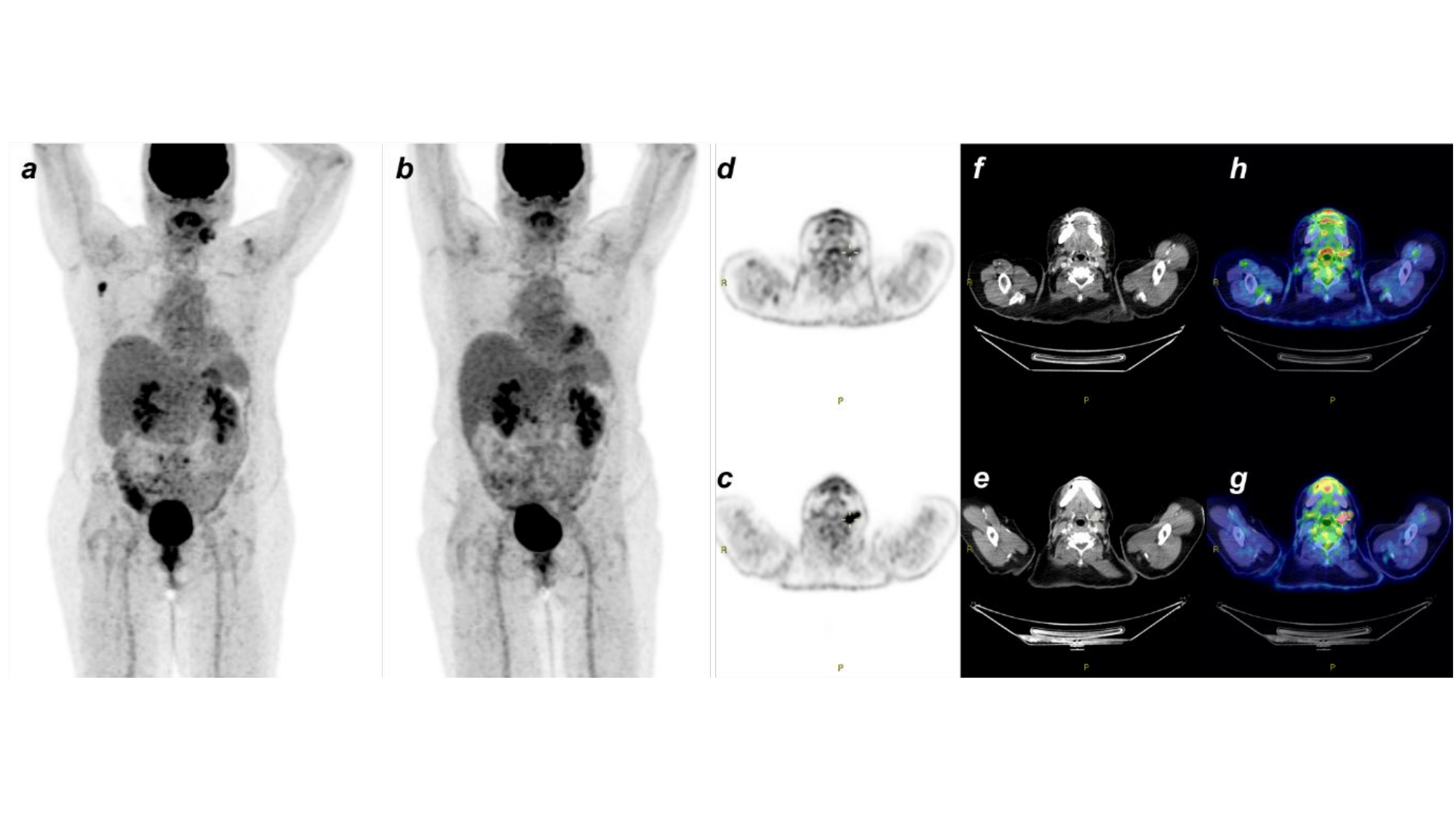

Supplement: Supplementary file 3 — Additional file 3: Fig. S3: Representative image of IgG4-RD patient without cardiovascular disease. Note the decreased 2-[18F]FDG uptake in the left submandibular gland and right axillary lymph nodes before (figures a, c, e, g) and after therapy with prednisolone (figures b, d, f, h) [file 13550_2023_972_MOESM3_ESM.pptx]
